# Autophagic Protein Beclin 1 Serves as an Independent Positive Prognostic Biomarker for Non-Small Cell Lung Cancer

**DOI:** 10.1371/journal.pone.0080338

**Published:** 2013-11-15

**Authors:** Weihua Zhou, Caifeng Yue, Jinyun Deng, Ronghuan Hu, Jie Xu, Long Feng, Qiongyu Lan, Wenfeng Zhang, Dexiang Ji, Jianbing Wu, Quentin Liu, Anwen Liu

**Affiliations:** 1 Department of Oncology, The Second Affiliated Hospital, Nanchang University, Nanchang, China; 2 State Key Laboratory of Oncology in South China, Cancer Center, Sun Yat-Sen University, Guangzhou, China; 3 Department of the Sixth Internal Medicine, Jiangxi Province Cancer Hospital, Nanchang, China; 4 Department of Infectious Disease, The First Affiliated Hospital, Nanchang University, Nanchang, China; 5 Department of Hematology, The First Affiliated Hospital, Nanchang University, Nanchang, China; West German Cancer Center, Germany

## Abstract

Beclin 1, a key regulator of autophagy, has been found to be aberrantly expressed in a variety of human malignancies. Herein, we employed immunohistochemistry (IHC) to detect the protein expression of Beclin 1 in non-small cell lung cancer (NSCLC) and paired normal adjacent lung tissues, and analyzed its clinicopathological/prognostic significance in NSCLC. Receiver operating characteristic (ROC) curve analysis was utilized to determine a cutoff point (>2 VS. ≤2) for Beclin 1 expression in a training set (n = 105). For validation, the ROC-derived cutoff value was subjected to analysis of the association of Beclin 1 with patients’ clinical characteristics and outcome in a testing set (n = 111) and the overall patient cohort (n = 216). Our data showed that Beclin 1 was significantly lower in NSCLC tissues compared with the adjacent normal tissues, negatively associating with tumor recurrence rate (65.8% VS 32.3%; *p* < 0.001). In the testing set and the overall patient cohort, low expression of Beclin 1 showed significantly inferior overall survival (OS) (*p* < 0.001) and progression-free survival (PFS) (*p* < 0.001) compared to high expression of Beclin 1. In the testing set and the overall patient cohort, the median duration of OS for patients with high and low expression of *Beclin 1* was 108 VS. 24.5 months (*p* < 0.001) and 108 VS. 28 months (*p* < 0.001), respectively. Furthermore, low expression of *Beclin 1* was also a poor prognostic factor within each stage of NSCLC patients. Multivariate analysis identified that *Beclin 1* was an independent prognostic factor for NSCLC. Our findings in the present study provided evidence that Beclin 1 may thus emerge as an independent prognostic biomarker in this tumor entity in the future.

## Introduction

Lung cancer accounts for 15% of cancer diagnoses, presenting as one of the leading causes of cancer-related death worldwide [1]. Non-small cell lung cancer (NSCLC) represents 80–85% of lung cancers [2]. Despite significant advances in surgical techniques and medical treatment, the 5-year survival rate in patients with NSCLC remains only 15% [2]. Difficulties of early diagnosis, high potential of metastasis and the occurrence of treatment resistance for advanced disease are responsible for the poor survival rate of NSCLC. Although much is known about the causal factors, clinical features, and pathogenesis of NSCLC, the molecular marker that has major clinical prognostic predictive value remains substantially limited. Many studies have demonstrated that multiple genetic alterations and epigenetic changes are responsible for the development and progression of NSCLC [3]. The presence of *KRAS* mutation represents the most common molecular change in NSCLC and has been shown to be associated with a poor prognosis [4]. Activation of PI3K/AKT pathway and loss of PTEN in NSCLC patients are associated with high grade and more advanced disease [5]. Furthermore, aberrant DNA methylation served as a marker for the early detection of lung cancer [6]. Promoter methylation of p16 is an indicator of poor prognosis of NSCLC [7]. Thus, identification of biologic markers might help to assess the prognosis more precisely or to decide more clearly the use of adjuvant therapy.

Autophagy refers to a cellular catabolic process that delivers cytoplasmic components to lysosomes for subsequent degradation or recycles long-lived proteins and organelles in order to maintain intracellular homeostasis within the cell [8]. There are three principle autophagic pathways, including microautophagy, chaperone-mediated autophagy (CMA), and macroautophagy [9]. Macroautophagy is the main subtype of autophagy (hereafter referred to as autophagy) [9]. Beclin 1, as the first identified mammalian autophagy effector, is essential for the initiation of autophagy by forming the Beclin 1-interacting complex, which consists of the BCL-2 family proteins, the class III phosphatidylinositol 3-kinase (VPS34), and ATG14L [10]. It becomes clear that Beclin 1 possesses a novel Bcl-2 homology region-3 (BH3) domain [11-13]. Bcl-2 family proteins can bind to the BH3 domain of Beclin 1 and inhibit Beclin 1-mediated autophagy by sequestering Beclin 1 away from hVps34 [11-13]. Modulation of Beclin 1 or BCL-2 by phosphorylation or ubiquitination can dissociate BCL-2 family members from Beclin 1 and activate VPS34 kinase activity, leading to an increase in autophagy [14]. Accordingly, apoptosis and autophagy may be co-regulated in the same directions. 

Recently, autophagy defect is reported to play a critical role in tumorigenesis and tumor progression [10,15-17]. Indeed, Beclin 1 has been reported to be mono-allelically decreased or deleted in human ovarian, breast, and prostate cancers [10]. Becn1^+/-^ tumors showed decreased autophagy, elevated cell stress and genome instability that ultimately accelerates mammary tumorigenesis [18]. Knockdown of *Beclin 1* in mice leads to inhibition of autophagy and subsequently a high incidence of spontaneous tumors, including lymphoma, liver and lung cancer [19,20]. Furthermore, reduced expression of Beclin 1 is found to be associated with the primary tumor growth in NSCLC [21]. These data indicates that *Beclin 1* serves as a haploinsufficient tumor-suppressor protein through its regulation of autophagy. Even so, autophagy seems to as a cytoprotective process and it was proposed as an alternative mechanism of drug resistance through promoting the tumor cell survival under unfavorable conditions. In lung cancer, several studies show that autophagy inhibition represents a promising approach to improve the efficacy of the treatment in patients with advanced non-small-cell lung cancer [22,23]. 

Herein, we analyzed the expression of autophagic protein *Beclin 1* in 216 NSCLC specimens and analyzed its correlation with clinicopathological factors of NSCLC patients. Our results showed that *Beclin 1*, as detected by immunohistochemistry, was significantly lower in NSCLC tissues compared with the adjacent normal tissues, and negatively associated with tumor recurrence rate. Low expression of Beclin 1 predicted an inferior OS and PFS in NSCLC. Furthermore, multivariate analysis revealed that *Beclin 1* was an independent prognostic factor for NSCLC.

## Materials and Methods

### Patients and tissue samples

A total of 244 primary NSCLC specimens from the archives of the Department of Pathology in the cancer center of Sun Yat-sen University (Guangzhou, China) were included in our study. All patients accepted curative surgery from 2001 to 2003. We further screened patients using a strict eligibility criteria protocol as following: microscopically confirmed NSCLC (including squamous cell carcinoma, adenocarcinoma and adenosquamous carcinoma); without any distant metastatic diseases; no prior chemotherapy or radiation therapy history; having over 5-year follow up period; including nonsmokers (daily cigarette consumption × years of smoking = 0) and smokers (former smokers and/or current smokers) [24]. Ultimately, 28 patients with loss of follow-up were not included in the present study, leading to 216 NSCLC patients subjecting to further clinical and survival analysis. Among the total patients (n=216), 116 (116/216, 53.7%) were censored as death during the 5 years of follow-up time (4 cases died from postoperative complications and 112 cases died from tumor progression).

Of the 216 NSCLC patients (median age, 47.0 year; range, 22–74 year), those with positive lymph node metastasis (n=100) were treated with four to six cycles of cisplatin + navelbine adjuvant chemotherapies after surgical resection, whereas patients with negative lymph node metastasis (n=116) did not receive adjuvant chemotherapies. Furthermore, no adjuvant radiotherapy was administered to any of the patients after surgery. The cohort of 216 NSCLC patients were then randomly divided into training set (n=105) and testing set (n=111) by computer (SPSS 17.0 software). Briefly, in the training set, there are 78 males and 27 females, with 38 cases for stage I, 31 cases for stage II and 36 cases for stage III. Meanwhile, there are 81 males and 30 females in the testing set, with 41 cases for stage I, 28 cases for stage II and 42 cases for stage III. The histological and stage type were determined according to the classification of NSCLC by WHO and International Union against Cancer Tumor-Node-Metastasis (TNM) staging system [25]. Written informed consent for the use of the tissues was obtained from all patients before surgery, and the study was approved by the Institute Research Ethics Committee of Sun Yat-Sen University.

### Tissue microarray construction and immunohistochemistry staining

Two representative core tissue biopsies (0.6-mm in diameter) were punched from represented NSCLC tissues and one cylinder with the same diameter from adjacent normal lung tissues. Multiple sections (5 µm thick) were cut from the TMA blocks and mounted on the microscope slides. One section from the TMA block was stained with Hematoxylin and Eosin (H&E) to confirm that the punches contained tumor.

Immunohistochemical staining was performed as described previously [26]. In brief, after deparaffinization, rehydration, antigen retrieval, and blocking, the TMA slides were incubated overnight at 4°C with a polyclonal antibody against human *Beclin 1* (1:200; Santa Cruz, SC-11427) in a moist chamber. Then slides were incubated in corresponding secondary antibodies (HRP-anti-Rabbit; Thermo Scientific, Cat. No. 31460) at room temperature for 30 min. Reaction products were visualized by staining with 3, 3-diaminobenzidine (3, 3-diaminobenzidine; Dako, Cat. No. K5007). After counterstained with hematoxylin, all the slides were dehydrated and stored. Negative controls were achieved by replacing the *Beclin 1* antibody with corresponding non-immune serum immunoglobulin. Our previously well-defined immunostaining- positive gastric cancer slides were used as positive control [27].

### Staining evaluation

The staining intensity and extent of *Beclin 1* was graded as described previously [27]. Briefly, the staining intensity was graded into negative (score 0), bordering (score 1), weak (score 2), moderate (score 3) and strong (score 4). In addition, the staining extent was also graded into five levels according to the percentage of cells with elevated Beclin 1 staining, including negative (score 0), 0–25% (score 1), 26–50% (score 2), 51–75% (score 3) and 76–100% (score 4). *Beclin 1* staining was assessed by two pathologists who were unaware of any clinical details related to the patients. The assessment was congruent in 85% of the cases, indicating a highly reproducible scoring system. In discrepant cases, a re-assessment was performed. The value was selected until both pathologists agreed with the result.

### Selection of a cutoff point for *Beclin 1* expression

Receiver operating characteristic (ROC) curve analysis is usually used in clinical oncology to evaluate and compare the sensitivity and specificity of diagnostic tests [28,29], or identify the threshold value above which a test result should be considered as positive for some outcome [28]. ROC curve analysis has been shown to be reproducible to evaluate IHC protein expression and to select biologically or clinically a relevant cut-off score for tumour positivity [27,28,30]. Herein, we used ROC curve analysis to select the cutoff point of *Beclin 1* for OS and PFS in the training set (n = 105). In brief, the score localized closest to the point at both maximum sensitivity and specificity (0.0, 1.0) was selected as the cutoff score of *Beclin 1*, leading to the greatest number of tumors which were correctly classified as having or not having the outcome. ROC curve analysis was facilitated by dichotomizing the features of patients’ outcome into survival (death VS. others (censored, alive or death from other causes)) and progression (local failure or distant metastasis).

### Follow up

All patients had follow-up records for over 5 years. After the completion of therapy, patients were observed at 3 month intervals during the first 3 years and at 6 month intervals thereafter. The latest follow-up was updated in September 2011. OS was defined as the time from the tumor resection to the date of death or when censored at the latest date if patients were still alive; PFS was defined as the time from the tumor resection to the date of disease relapse/progression or the date of death or when censored at the latest date. 

### Statistical analysis

ROC analysis was used to get an optimal cutoff point of *Beclin 1* expression for survival analysis in the training set (n = 105). For validation, the relationship between *Beclin 1* expression and OS, PFS were evaluated in the testing set (n = 111) and the overall patient cohort (n = 216). Relationship between *Beclin 1* expression and clinicopathological variables were analyzed by the chi-square test or Fisher’s exact test. Kaplan-Meier analysis was employed to evaluate the relationship between *Beclin 1* expression and OS and PFS. Differences in survival probabilities between patient subsets were assessed by the log-rank tests. The multivariate Cox proportional hazards model was utilized to estimate the hazard ratios and 95% confidence intervals for patient outcome. All *p* values quoted were two sided and *p* < 0.05 was considered statistically significant. Statistical analysis was performed using SPSS v. 17.0 (SPSS, Inc, Chicago, IL).

## Results

### *Beclin 1* Expression in NSCLC and normal adjacent tissues

**Figure 1:**
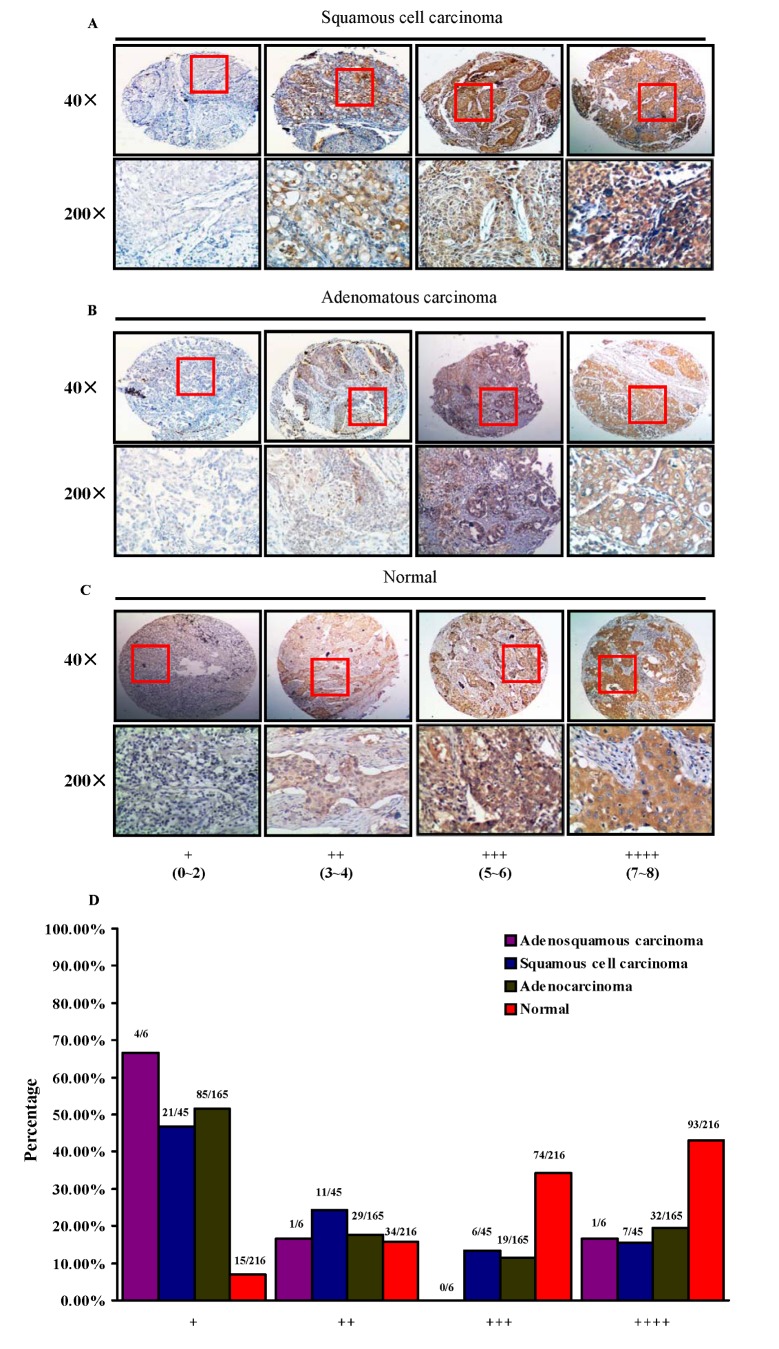
IHC analysis of *Beclin 1* expression in human NSCLC and normal adjacent tissues. (A-C), NSCLC tissue arrays containing normal lung and tumor tissues were stained for *Beclin 1* expression. Stained normal and tumor tissues were classified into four groups (+ to ++++) according to the staining intensity and extension of each tissue. (D), percentage of normal or tumor tissues in each staining group. Tissue samples with different staining intensity and extension were grouped and tabulated.

Immunohistochemistry was employed to examine the protein expression of *Beclin 1* in primary NSCLC specimens and normal adjacent tissues. Immunoreactivity of *Beclin 1* was observed primarily in the cytoplasm (Figure 1 and Figure S1). Based on the combination of intensity of staining and the extent, the samples were classified into four groups, from group 1 with nearly negative staining (+), group 2 with the weak staining (++), group 3 with moderate staining (+++), to group 4 with the strong staining (++++; Figure 1A-C). As shown in Figure 1, high expression of *Beclin 1* is mainly found in normal lung tissues, manifesting the large number of samples with group 3 (74 of 216, 34.3%, Figure 1D) and group 4 (93 of 216, 43.1%, Figure 1D), whereas lung tumor tissues, regardless of tumor subtypes, showed moderate *Beclin 1* staining (Figure 1A, B, D)

**Figure 2:**
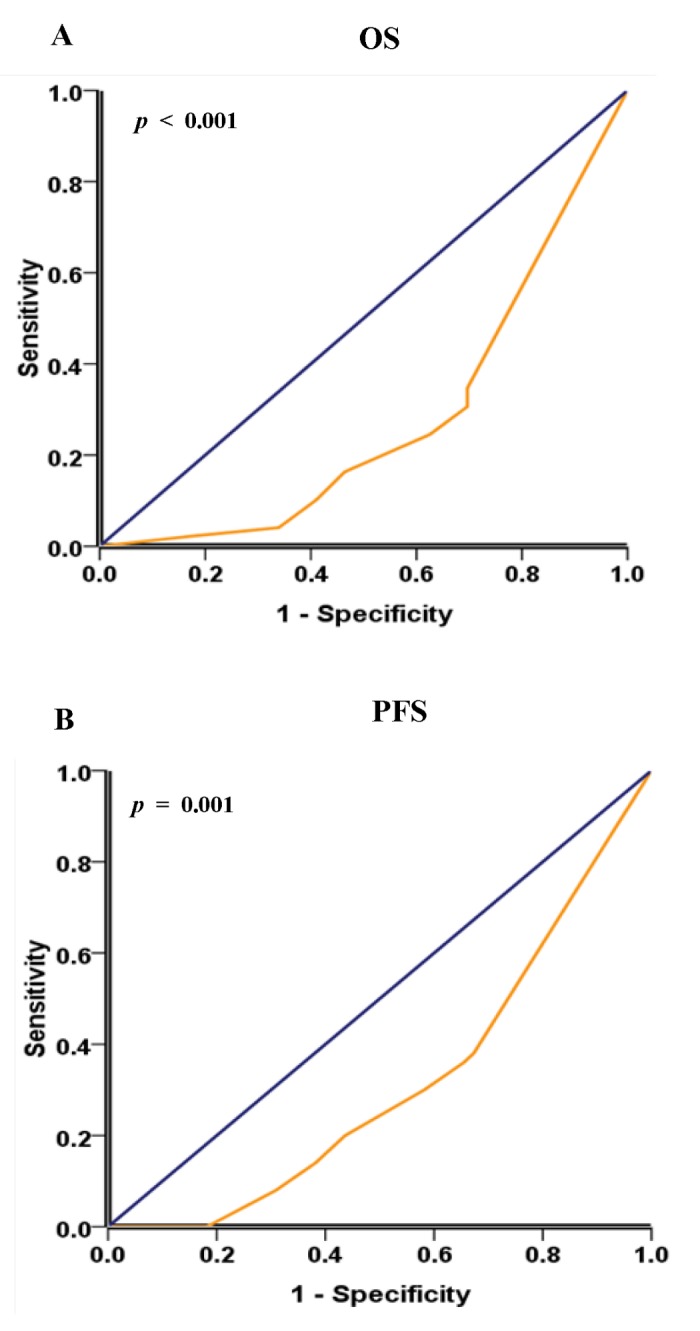
ROC curves analysis of *Beclin 1* cutoff score. (A) and (B) showed the *Beclin 1* cutoff points for overall survival and progression-free survival in the training set. At each immunohistochemical score, the sensitivity and specificity for the outcome being studied was plotted, thus generating a ROC curve. The cutoff point of *Beclin 1* for overall survival and progression-free survival was 2.7 and 2.9 respectively.

For further survival analysis and avoid the problems of multiple cutpoint selection, ROC curve analysis was performed to determine a reasonable cutoff point of *Beclin 1* in the training set (n = 105). The *Beclin 1* cutoff point for OS and PFS in the training set was 2.7 (*p* < 0.001, Figure 2A) and 2.9 (*p* = 0.001, Figure 2B), respectively. A score of 2 (>2 VS. ≤2) for *Beclin 1* expression was selected as the uniform cutoff point to distinguish NSCLC patients as high or low expression of *Beclin 1*. 

### *Beclin 1* expression and clinical features

The clinical features of patients, including age, gender, smoking history, CEA level, initial clinical stage, histology, differentiation, tumor stage, lymph node metastasis, recurrence, and *Beclin 1* expression, were summarized in Table 1. The *Beclin 1* cutoff score of 2 derived from the training set successfully segregated the testing set into two subgroups. In brief, patients with a cutoff score >2 are regarded as Beclin 1 high expression (50/111, 45.0%) and those with a score ≤2 are viewed as Beclin 1 low expression (61/111, 55.0%). Low expression of *Beclin 1* was mainly detected in patients with positive recurrence (78/117, 65.8% in patients with positive recurrence VS. 32/99, 32.3% in patients with negative recurrence, *p* < 0.001). Furthermore, correlation analysis demonstrated that *Beclin 1* expression was negatively correlated with tumor recurrence rate in both sets (*p* < 0.001 in the training set and *p* = 0.001 in the testing set; Table 1). In addition, *Beclin 1* expression also negatively correlated with the initial tumor stage (*p* < 0.001, Table 1). Low expression of *Beclin 1* was associated with metastatic spread to the lymph nodes (*p* = 0.001, Table 1) in the training set, and correlated with differentiation (*p* = 0.020, Table 1) and CEA level (*p* = 0.002, Table 1) in the testing set. We could not show any correlation between Beclin 1 expression and other patient characteristics including age, gender, smoking history etc.

**Table 1:** Association of *Beclin 1* expression with patient’s clinicopathologic characteristics in NSCLC.

**Variable**	**All cases**	**Training Set (n = 105)**			**Testing Set (n = 111)**		
		**High**	**Low**	***P***	**High**	**Low**	***P***
**Age (years)**							
≥ 60.0	105	26	21		25	33	
< 60.0	111	30	28	0.713	25	28	0.667
**Gender**							
Male	159	43	35		34	47	
Female	57	13	14	0.531	16	14	0.285
**Smoking history**							
Yes	128	35	27		27	39	
No	88	21	22	0.442	23	22	0.289
**CEA (ng/ml)**							
> 5.0	97	22	24		15	36	
≤ 5.0	119	34	25	0.318	35	25	0.002
**Initial clinical stage**							
i	79	27	11		23	18	
II	59	20	11	0.000	12	16	0.169
III	78	9	27		15	27	
**Histology**							
Squamous cell carcinoma	45	14	9		10	12	
Adenocarcinoma	165	42	40	0.412	38	45	0.839
Adenosquamous carcinoma	6	0	0		2	4	
**Differentiation**							
Well	44	12	7		10	15	
Moderately	72	23	15	0.192	22	12	0.020
Poorly	100	21	27		18	34	
**Tumor stage**							
T_1_+T_2_	160	44	31		42	43	
T_3_+T_4_	56	12	18	0.083	8	18	0.095
**Lymph node metastasis**							
Negative	116	40	19		29	28	
Positive	100	16	30	0.001	21	33	0.205
**Recurrence**							
Positive	117	17	33		22	45	
Negative	99	39	16	0.000	28	16	0.001

### *Beclin 1* expression and survival analysis: univariate survival analysis

**Figure 3:**
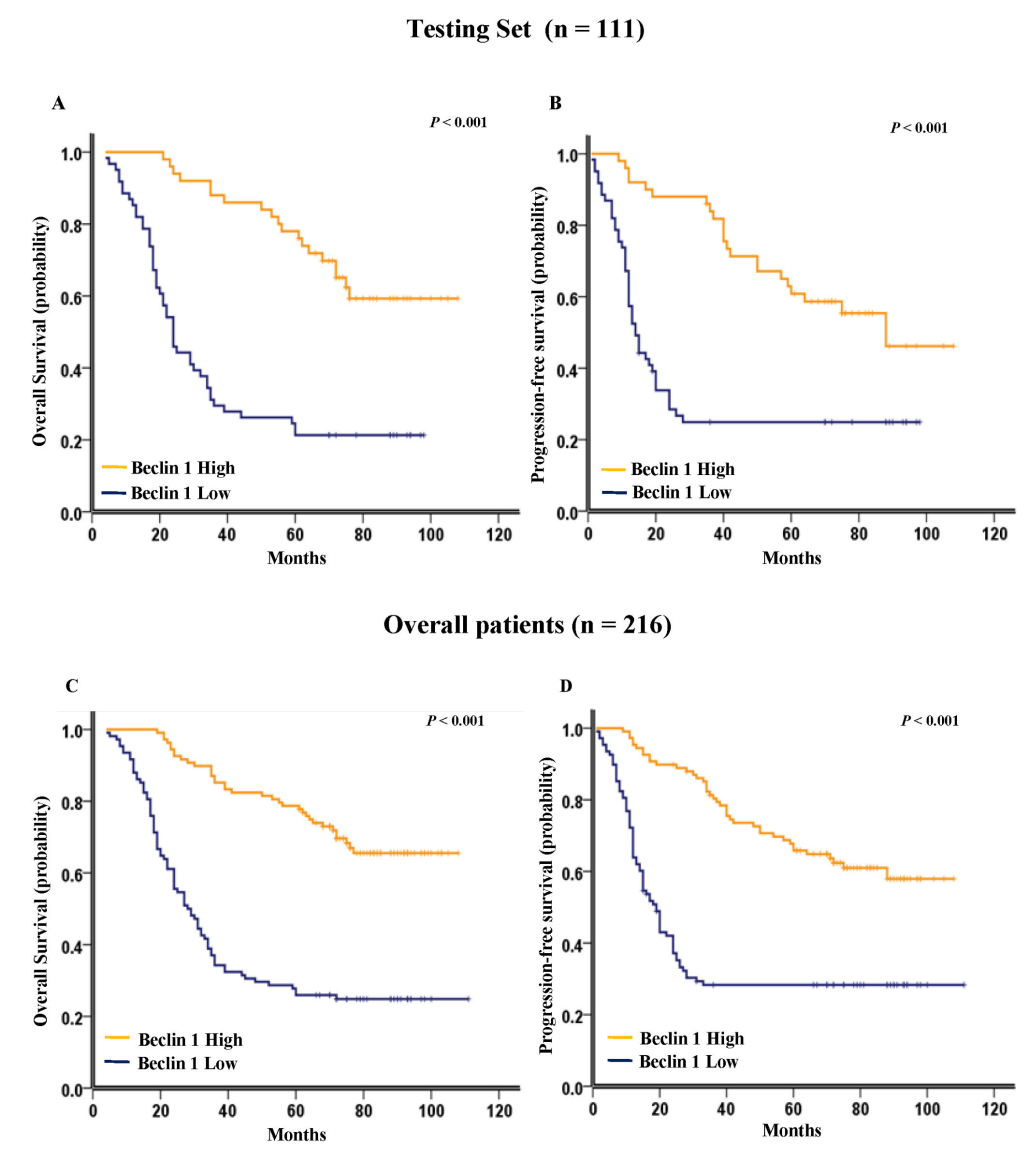
Kaplan-Meier survival analysis of *Beclin 1* expression in the testing set and the overall patient cohort. (A) Low expression of *Beclin 1* was closely correlated with poor overall survival and (B) progression-free survival in the testing set (n = 111). (C) Patients with lower *Beclin 1* expression also acquired an inferior overall survival and (D) progression-free survival in the the overall patient cohort (n = 216). In the testing set and the overall patient cohort, the median duration of overall survival for patients with high and low expression of *Beclin 1* was 108 VS. 24.5 months (*p* < 0.001) and 108 VS.28 months (*p* < 0.001), respectively.

**Figure 4:**
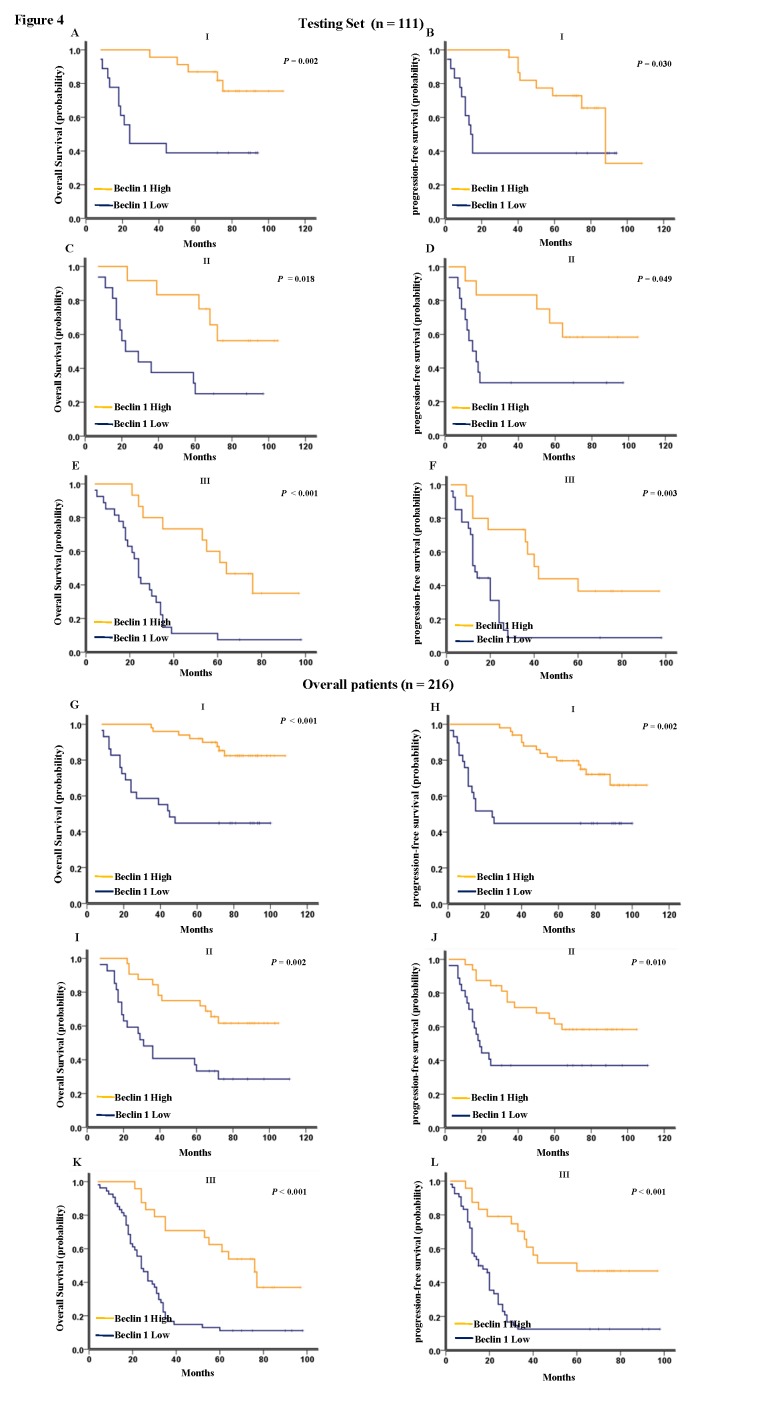
Kaplan-Meier survival analysis of *Beclin 1* expression in subsets of NSCLC patients. (A) Probability of overall survival and (B) progression-free survival of NSCLC patients with stage i in the testing set: low expression, n = 18; high expression, n = 23. (C) Probability of overall survival and (D) progression-free survival of NSCLC patients with stage ii in the testing set: low expression, n = 16; high expression, n = 12. (E) Probability of overall survival and (F) progression-free survival of NSCLC patients with stage iii in the testing set: low expression, n = 27; high expression, n = 15. (G) Probability of overall survival and (H) progression-free survival of NSCLC patients with stage Ⅰin the the overall patient cohort: low expression, n = 29; high expression, n = 50. (I) Probability of overall survival and (J) progression-free survival of NSCLC patients with stage ii in the the overall patient cohort: low expression, n = 27; high expression, n = 32. (K) Probability of overall survival and (L) progression-free survival of NSCLC patients with stage iii in the the overall patient cohort: low expression, n = 54; high expression, n = 24.

As shown in Figure 3, Kaplan-Meier analysis showed that low expression of *Beclin 1* strongly predicted an inferior OS and PFS in the testing set (*p* < 0.001 for both OS and PFS, Figure 3A and 3B) and the overall patient cohort (*p* < 0.001 for both OS and PFS, Figure 3C and 3D). In the testing set and the overall patient cohort, the median duration of overall survival for patients with high and low expression of *Beclin 1* was 108 VS. 24.5 months (*p* < 0.001) and 108 VS.28 months (*p* < 0.001), respectively. Further analysis was performed between *Beclin 1* expression and subsets of NSCLC patients within each clinical stage. Low expression of *Beclin 1* also served as a poor prognostic factor in each stage of NSCLC patients in the testing set: stage I (*p* = 0.002 for OS and *p* = 0.030 for PFS, Figure 4A and 4B), stage II (*p* = 0.018 for OS and *p* = 0.049 for PFS, Figure 4C and 4D), and stage III (*p* < 0.001 for OS and *p* = 0.003 for PFS, Figure 4E and 4F). For the the overall patient cohort, results were similar to those found in the testing set (Figure 4G-L).

### Multivariate Cox regression analysis

For the purpose of avoiding the influence caused by univariate analysis, the expression of *Beclin 1* as well as other parameters was examined in multivariate Cox analysis in the testing set and the overall patient cohort (Table 2 and Table 3). As shown in Table 2, *Beclin 1* was an independent biomarker to predict the prognosis of OS (hazard ratio, 4.235; 95% CI, 2.382 to 7.528; *p* < 0.001, Table 2) and PFS (hazard ratio, 3.420; 95% CI, 1.936 to 6.042; *p* < 0.001, Table 2) in the testing set. Similar observation was also found in the overall patient cohort (for OS, hazard ratio, 3.721; 95% CI, 2.453 to 5.645; *p* < 0.001, Table 3; for PFS, hazard ratio, 2.987; 95% CI, 2.004 to 4.453; *p* < 0.001, Table 3). Furthermore, CEA level, lymph node metastasis, smoking history, and age were also identified as independent prognostic parameters for OS and/or PFS in the testing set and/or the overall patient cohort (Table 2, Table 3). However, initial clinical stage, as a well-characterized independent prognostic factor, was not detected as independent prognostic factor in our results.

**Table 2:** Results of multivariate Cox proportional-hazards analysis in the testing set (n = 111).

**Variable**	**For death**			**For progression-free survival**		
	**Hazard Ratio**	**95% confidence interval**	***P***	**Hazard Ratio**	**95% confidence interval**	***P***
**Age (years) ≥ 60.00 (VS. < 60.0)**	1.266	(0.734 to 2.185)	0.396	1.053	(0.613 to 1.808)	0.852
**Gender Male (VS. Female)**	3.289	(1.336 to8.096)	0.010	3.449	(1.535 to 7.747)	0.003
**Smoking history Yes (VS. No )**	4.643	(1.958 to 11.009)	0.000	2.443	(1.158 to 5.152)	0.019
**CEA (ng/ml) > 5 (VS. ≤ 5)**	2.556	(1.516 to 4.309)	0.000	2.133	(1.271 to 3.581)	0.004
**Initial clinical stage**						
**I**	0.998	(0.277 to 3.603)	0.998	1.063	(0.267 to 4.231)	0.931
**II**	0.785	(0.340 to 1.813)	0.571	0.928	(0.387 to 2.228)	0.868
**III**	1	1		1	1	
**Histology**						
**Squamous cell carcinoma**	1.558	(0.443 to 5.478)	0.490	1.195	(0.405 to 3.523)	0.747
**Adenocarcinoma**	1.474	(0.378 to 5.742)	0.576	0.640	(0.179 to 2.293)	0.493
**Adenosquamous carcinoma**	1	1		1	1	
**Differentiation**						
**Well**	0.886	(0.414 to 1.897)	0.756	0.538	(0.258 to 1.121)	0.098
**Moderately**	1.159	(0.578 to 2.324)	0.677	0.641	(0.315 to 1.303)	0.219
**Poorly**	1	1		1	1	
**Tumor stage T_4_ + T_3_ (VS. T_2_ + T_1_)**	1.091	(0.583 to 2.042)	0.785	1.359	(0.715 to 2.585)	0.350
**Lymph node metastasis Positive (VS. Negative)**	2.448	(1.428 to 4.199)	0.001	1.854	(1.098 to 3.130)	0.021
***Beclin 1* Low (VS. High )**	4.235	(2.382 to 7.528)	0.000	3.420	(1.936 to 6.042)	0.000

**Table 3:** Results of multivariate Cox proportional-hazards analysis in the overall patient cohort (n = 216).

**Variable**	**For death**			**For progression-free survival**		
	**Hazard Ratio**	**95% confidence interval**	***P***	**Hazard Ratio**	**95% confidence interval**	***P***
**Age (years) ≥ 60.00 (VS. < 60.0)**	1.375	(0.951 to 1.988)	0.091	1.039	(0.708 to 1.524)	0.846
**Gender Male (VS. Female)**	1.422	(0.794 to 2.546)	0.236	1.285	(0.744 to 2.219)	0.369
**Smoking history Yes (VS. No )**	1.493	(1.021 to 2.184)	0.039	1.105	(0.637 to 1.917)	0.723
**CEA (ng/ml) > 5 (VS. ≤ 5)**	2.186	(1.484 to 3.220)	0.000	2.043	(1.395 to 2.992)	0.000
**Initial clinical stage**						
**I**	0.758	(0.286 to 2.012)	0.578	0.969	(0.354 to 2.652)	0.951
**II**	0.853	(0.467 to 1.558)	0.605	0.928	(0.494 to 1.742)	0.816
**III**	1	1		1	1	
**Histology**						
**Squamous cell carcinoma**	1.239	(0.379 to 4.047)	0.723	0.987	(0.354 to 2.746)	0.979
**Adenocarcinoma**	1.070	(0.305 to 3.763)	0.916	0.632	(0.199 to 1.950)	0.417
**Adenosquamous carcinoma**	1	1		1	1	
**Differentiation**						
**Well**	0.765	(0.434 to 1.347)	0.353	0.640	(0.369 to 1.110)	0.112
**Moderately**	0.959	(0.619 to 1.485)	0.850	0.931	(0.606 to 1.430)	0.745
**Poorly**	1	1		1	1	
**Tumor stage T_4_ + T_3_ (VS. T_2_ + T_1_)**	1.122	(0.701 to 1.794)	0.631	1.195	(0.678 to 1.814)	0.477
**Lymph node metastasis Positive (VS. Negative)**	2.576	(1.725 to 3.847)	0.000	1.862	(1.262 to 2.746)	0.002
***Beclin 1* Low (VS. High )**	3.721	(2.453 to 5.645)	0.000	2.987	(2.004 to 4.453)	0.000

## Discussion

Autophagy, which is highly conserved from yeast to human, is an essential catabolic pathway for the degradation of cellular components within the lysosome [8]. Different from the Ubiquitin-Proteasome System (UPS), which directly degrades a variety of regulatory proteins in the cytoplasm or nucleus, autophagy targets a wide spectrum of substrates including protein aggregates, long-lived proteins, or damaged organelles towards lysosomes for subsequent degradation. Autophagy involves in a series of normal and pathological processes. Recently, autophagy emerged as a key regulator of multiple aspects of cancer biology, including the initiation and progression of cancer, as well as the influence on the effectiveness of therapeutic interventions of this disease [31,32]. Autophagy may show an anticarcinogenic function in primary cells by safeguarding against metabolic stress through the homeostatic turnover of mitochondria and the elimination of protein aggregates. *Beclin 1*, as a key regulator of autophagy, gets much attention in recent years due to its involvement in carcinogenesis and cancer progression. Clinical studies have associated poor prognosis and aggressive tumor phenotypes with aberrant expression of *Beclin 1* in tumor tissues [27,33-36]. Furthermore, Beclin 1-dependent autophagic function has been shown to be suppressed in human cancer through activating AKT [37].

Recently, decreased expression of *Beclin 1* and *LC3* were detected in human lung cancer tissues, indicating a possible role in the pathogenesis of lung cancer. However, none of the afore-mentioned studies addressed the prognostic role of Beclin 1 in lung cancer so far [38]. Additionally, another study showed that *Beclin 1* expression was inversely associated with tumor size and primary tumor stage (pT) in lung squamous cell carcinoma and adenocarcinoma, but shows no relationship with overall survival [39]. Here, to avoid the problems of multiple cutpoint selection and get an unbiased *Beclin 1* cutoff point for clinical implication, we used ROC curve analysis as an alternative method in the selection and validation of cut-off scores to determine the most clinically relevant threshold for immunohistochemical tumour positivity. We analyzed the clinicopathological/prognostic significance of *Beclin 1* in a total number of 216 NSCLC patients, which consists of cohort I (n = 105) for training set and cohort II (n = 111) for testing set. The ROC-derived cutoff value was subjected to analysis of the association of *Beclin 1* with patients’ clinical characteristics and outcome in a testing set (n = 111) and the overall patient cohort (n = 216). 

Consequently, *Beclin 1* expression, was mainly found to be low in patients with disease relapse (Table 1), indicating that *Beclin 1* might be involved in NSCLC progression. Furthermore, Kaplan-Meier survival analysis showed that low expression of *Beclin 1* predicted a significant OS and PFS disadvantage over high expression of *Beclin 1* subgroup in the testing set and the overall patient cohort (Figure 3). In addition, low Beclin 1 expression remained a poor prognostic factor in each stage of NSCLC patients (Figure 4), emerging as an independent prognostic factor for NSCLC (Table 2, Table 3). However, initial clinical stage was not detected as independent prognostic factor in our results. Anyhow, there is a trend for better overall survival in the lower clinical stages, which account for small cases and are not statistically significant.

Considering the prognostic impact of *Beclin 1* protein in different human cancers, reports have drawn complicated conclusions. Previously, a number of studies have documented that *Beclin 1* expression is down-regulated in breast cancer, liver cancer, cervical and ovarian cancer, associating with an inferior prognosis [33,34,36]. More recently, we further demonstrated that low expression of *Beclin 1* predicted a poor prognosis in gastric cancer [27]. However, on the contrary, an elevated *Beclin 1* expression was strongly correlated with poor OS and PFS in nasopharyngeal carcinoma (NPC) [40]. With regard to the distinct prognostic impact of *Beclin 1* protein in various types of tumors, we speculate that it might be dependent on intrinsic properties of the tumor type, as well as the nature of the therapeutic regimen in various types of human cancers. For example, the patients of breast cancer patients are submitted to a postoperative treatment schedule of radiotherapy or chemotherapy, and sometimes with tamoxifen therapy according to the hormone receptors, and NPC patients are more radiosensitive than other malignant tumors. However, some NSCLC patients with early stage accept curative recection without radiotherapy or chemotherapy. 

Consistent with most of the previous studies, our results showed that *Beclin 1* protein was down-regulated in NSCLC and low expression of Beclin 1 was significantly associated with poor prognosis, indicating the potential tumor suppressing role of *Beclin 1* in this tumor. The underlying mechanism(s) of *Beclin 1* to suppress tumorigenesis and tumor progression might be attributed to its multi-functions. First of all, expression of *Beclin 1* stabilizes chromosome structure thereby prohibiting the process of carcinogenesis and tumor progression [20]. Furthermore, autophagy helps to eliminate damaged organelles, and then prevents oxidative stress from the damaged organelles [17]. In addition, *Beclin 1* down-regulates the proliferation of cells, delays cell cycle progression, and induces autophagy and differentiation [17]. Conclusionly, our findings showed that low expression of Beclin 1 help to identify patients that are at high risk of progression, and shows clinical value in predicting the prognosis of NSCLC, emerging as a potential independent biomarker for NSCLC.

## Supporting Information

Figure S1 **Whole picture of TMA with *Beclin 1* staining.**
(TIF)

## References

[1] Jemal A, Siegel R, Ward E, Hao Y, Xu J et al. (2008) Cancer statistics. CA Cancer J Clin 58(2): 71-96. doi:10.3322/CA.2007.0010. PubMed: 18287387. 18287387

[2] Kamangar F, Dores GM, Anderson WF (2006) Patterns of cancer incidence, mortality, and prevalence across five continents: defining priorities to reduce cancer disparities in different geographic regions of the world. J Clin Oncol 24(14): 2137-2150. doi:10.1200/JCO.2005.05.2308. PubMed: 16682732. 16682732

[3] Risch A, Plass C (2008) Lung cancer epigenetics and genetics. Int J Cancer 123(1): 1-7. doi:10.1002/ijc.23605. PubMed: 18425819. 18425819

[4] Roberts PJ, Stinchcombe TE (2013) *KRAS* Mutation: Should We Test for It, and Does It Matter? J Clin Oncol 31(8): 1112-1121. doi:10.1200/JCO.2012.43.0454. PubMed: 23401440. 23401440

[5] Scrima M, De Marco C, Fabiani F, Franco R, Pirozzi G, Rocco G et al. (2012) Signaling networks associated with AKT activation in non-small cell lung cancer (NSCLC): new insights on the role of phosphatydil-inositol-3 kinase. PLOS ONE 7(2): e30427. doi:10.1371/journal.pone.0030427. PubMed: 22363436. 22363436 PMC3281846

[6] Lokk K, Vooder T, Kolde R, Välk K, Võsa U et al. (2012) Methylation markers of early- stage non-small cell lung cancer. PLOS ONE 7 (6): e39813. doi:10.1371/journal.pone.0039813. PubMed: 22768131. 22768131 PMC3387223

[7] Lou-Qian Z, Rong Y, Ming L, Xin Y, Feng J et al. (2013) The prognostic value of epigenetic silencing of p16 gene in NSCLC patients: a systematic review and meta-analysis. PLOS ONE 8(1): e54970. doi:10.1371/journal.pone.0054970. PubMed: 23372805. 23372805 PMC3555860

[8] Kroemer G, Jäättelä M (2005) Lysosomes and autophagy in cell death control. Nat Rev Cancer 5(11): 886-897. doi:10.1038/nrc1738. PubMed: 16239905. 16239905

[9] Mizushima N, Levine B, Cuervo AM, Klionsky DJ (2008) Autophagy fights disease through cellular self-digestion. Nature 451(7182): 1069-1075. doi:10.1038/nature06639. PubMed: 18305538. 18305538 PMC2670399

[10] Aita VM, Liang XH, Murty VV, Pincus DL, Yu W et al. (1999) Cloning and genomic organization of beclin 1, a candidate tumor suppressor gene on chromosome 17q21. Genomics 59(1): 59-65. doi:10.1006/geno.1999.5851. PubMed: 10395800. 10395800

[11] Maiuri MC, Le Toumelin G, Criollo A, Rain JC, Gautier F et al. (2007) Functional and physical interaction between Bcl-X(L) and a BH3-like domain in Beclin-1. EMBO J 26(10): 2527-2539. doi:10.1038/sj.emboj.7601689. PubMed: 17446862. 17446862 PMC1868901

[12] Oberstein A, Jeffrey PD, Shi Y (2007) Crystal structure of the Bcl-XL-Beclin 1 peptide complex: Beclin 1 is a novel BH3-only protein. J Biol Chem 282(17): 13123-13132. doi:10.1074/jbc.M700492200. PubMed: 17337444. 17337444

[13] Pattingre S, Tassa A, Qu X, Garuti R, Liang XH et al. (2005) Bcl‑2 antiapoptotic proteins inhibit Beclin 1-dependent autophagy. Cell 122(6): 927-939. doi:10.1016/j.cell.2005.07.002. PubMed: 16179260. 16179260

[14] Abrahamsen H, Stenmark H, Platta HW (2012) Ubiquitination and phosphorylation of Beclin 1 and its binding partners: Tuning class III phosphatidylinositol 3-kinase activity and tumor suppression. FEBS Lett 586(11): 1584-1591. doi:10.1016/j.febslet.2012.04.046. PubMed: 22673570. 22673570

[15] Rosenfeldt MT, Ryan KM (2011) The multiple roles of autophagy in cancer. Carcinogenesis 32(7): 955-963. doi:10.1093/carcin/bgr031. PubMed: 21317301. 21317301 PMC3128556

[16] Mathew R, Karantza-Wadsworth V, White E (2007) Role of autophagy in cancer. Nat Rev Cancer 7(12): 961-967. doi:10.1038/nrc2254. PubMed: 17972889. 17972889 PMC2866167

[17] Liang XH, Jackson S, Seaman M, Brown K, Kempkes B et al. (1999) Induction of autophagy and inhibition of tumorigenesis by beclin 1. Nature 402(6762): 672-676. doi:10.1038/45257. PubMed: 10604474. 10604474

[18] Mathew R, Kongara S, Beaudoin B, Karp CM, Bray K et al. (2007) Autophagy suppresses tumor progression by limiting chromosomal instability. Genes Dev 21(11): 1367-1381. doi:10.1101/gad.1545107. PubMed: 17510285. 17510285 PMC1877749

[19] Qu X, Yu J, Bhagat G, Furuya N, Hibshoosh H et al. (2003) Promotion of tumorigenesis by heterozygous disruption of the beclin 1 autophagy gene. J Clin Invest 112(12): 1809-1820. doi:10.1172/JCI200320039. PubMed: 14638851. 14638851 PMC297002

[20] Yue Z, Jin S, Yang C, Levine AJ, Heintz N (2003) Beclin 1, an autophagy gene essential for early embryonic development, is a haploinsufficient tumor suppressor. Proc Natl Acad Sci U_S_A 100(25): 15077-15082. doi:10.1073/pnas.2436255100. PubMed: 14657337. 14657337 PMC299911

[21] Won KY, Kim GY, Lim SJ, Kim YW ( 1 2012) Decreased Beclin-1 expression is correlated with the growth of the primary tumor in patients with squamous cell carcinoma and adenocarcinoma of the lung. Hum Pathol 1; 43(1): 62-68. doi:10.1016/j.humpath.2011.04.007. PubMed: 21777947. 21777947

[22] Han W, Pan H, Chen Y, Sun J, Wang Y et al. (2011) EGFR tyrosine kinase inhibitors activate autophagy as a cytoprotective response in human lung cancer cells. PLOS ONE 6(6): e18691. doi:10.1371/journal.pone.0018691. PubMed: 21655094. 21655094 PMC3107207

[23] Pan X, Zhang X, Sun H, Zhang J, Yan M et al. (2013) Autophagy inhibition promotes 5-fluorouraci-induced apoptosis by stimulating ROS formation in human non-small cell lung cancer A549 cells. PLOS ONE 8(2): e56679. doi:10.1371/journal.pone.0056679. PubMed: 23441212. 23441212 PMC3575481

[24] Zhang B, Zhu W, Yang P, Liu T, Jiang M et al. (2011) Cigarette smoking and p16INK4α gene promoter hypermethylation in non-small cell lung carcinoma patients: ameta-analysis. PLOS ONE 6(12): e28882. doi:10.1371/journal.pone.0028882. PubMed: 22174919. 22174919 PMC3236763

[25] Sobin LH, Fleming ID (1997) TNM Classification of Malignant Tumors, fifth edition; Union Internationale Contre le Cancer and The American Joint Committee on Cancer. Cancer 80(9) (1803-4) 10.1002/(sici)1097-0142(19971101)80:9<1803::aid-cncr16>3.0.co;2-9 9351551

[26] Zhou WH, Tang F, Xu J, Wu X, Feng ZY et al. (2011) Aberrant upregulation of 14-3-3ơ expression serves as an inferior prognostic biomarker for gastric cancer. BMC Cancer 11: 397. doi:10.1186/1471-2407-11-397. PubMed: 21933426. 21933426 PMC3184120

[27] Zhou WH, Tang F, Xu J, Wu X, Yang SB et al. (2012) Low expression of Beclin 1, associated with high Bcl-xL, predicts a malignant phenotype and poor prognosis of gastric cancer. Autophagy 8(3): 389-400. doi:10.4161/auto.18641. PubMed: 22240664. 22240664

[28] Hanley JA (1989) Receiver operating characteristic (ROC) methodology: the state of the art. Crit Rev Diagn Imaging 29(3): 307-335. PubMed: 2667567. 2667567

[29] Lind PA, Wennberg B, Gagliardi G, Rosfors S, Blom-Goldman U et al. (2006) ROC curves and evaluation of radiation-induced pulmonary toxicity in breast cancer. Int J Radiat Oncol Biol Phys 64(3): 765-770. doi:10.1016/j.ijrobp.2005.08.011. PubMed: 16257129. 16257129

[30] Zlobec I, Steele R, Terracciano L, Jass JR, Lugli A (2007) Selecting immunohistochemical cut-off scores for novel biomarkers of progression and survival in colorectal cancer. J Clin Pathol 60(10): 1112-1116. doi:10.1136/jcp.2006.044537. PubMed: 17182662. 17182662 PMC2014838

[31] Choi AM, Ryter SW, Levine B (2013) Autophagy in Human Health and Disease. N Engl J Med 368(19): 1845-1846. doi:10.1056/NEJMc1303158. PubMed: 236566592365665823406030. 23656658

[32] Kimmelman AC (2011) The dynamic nature of autophagy in cancer. Genes Dev 25(19): 1999-2010. doi:10.1101/gad.17558811. PubMed: 21979913. 21979913 PMC3197199

[33] Shi YH, Ding ZB, Zhou J, Qiu SJ, Fan J (2009) Prognostic significance of Beclin 1-dependent apoptotic activity in hepatocellular carcinoma. Autophagy 5(3): 380-382. doi:10.4161/auto.5.3.7658. PubMed: 19145109. 19145109

[34] Shen Y, Li DD, Wang LL, Deng R, Zhu XF (2008) Decreased expression of autophagy-related proteins in malignant epithelial ovarian cancer. Autophagy 4(8): 1067-1068. PubMed: 18776739. 18776739 10.4161/auto.6827

[35] Miracco C, Cosci E, Oliveri G, Luzi P, Pacenti L et al. (2007) Protein and mRNA expression of autophagy gene Beclin 1 in human brain tumours. Int J Oncol 30(2): 429-436. PubMed: 17203225. 17203225

[36] Karantza-Wadsworth V, White E (2007) Role of autophagy in breast cancer. Autophagy 3(6): 610-613. PubMed: 17786023. 17786023 10.4161/auto.4867 PMC2859167

[37] Wang RC, Wei Y, An Z, Zou Z, Xiao G et al. (2012) Akt-mediated regulation of autophagy and tumorigenesis through Beclin 1 phosphorylation. Science 338(6109): 956-959. doi:10.1126/science.1225967. PubMed: 23112296. 23112296 PMC3507442

[38] Jiang ZF, Shao LJ, Wang WM, Yan XB, Liu RY (2012) Decreased expression of Beclin-1 and LC3 in human lung cancer. Mol Biol Rep 39(1): 259-267. doi:10.1007/s11033-011-0734-1. PubMed: 21556768. 21556768

[39] Won KY, Kim GY, Lim SJ, Kim YW (2012) Decreased Beclin-1 expression is correlated with the growth of the primary tumor in patients with squamous cell carcinoma and adenocarcinoma of the lung. Hum Pathol 43(1): 62-68. doi:10.1016/j.humpath.2011.04.007. PubMed: 21777947. 21777947

[40] Wan XB, Fan XJ, Chen MY, Xiang J, Huang PY et al. (2010) Elevated Beclin 1 expression is correlated with HIF-1alpha in predicting poor prognosis of nasopharyngeal carcinoma. Autophagy 6(3): 395-404. doi:10.4161/auto.6.3.11303. PubMed: 20150769. 20150769

