# Supplementary figures and images for: Autophagic Protein Beclin 1 Serves as an Independent Positive Prognostic Biomarker for Non-Small Cell Lung Cancer
Source: PLoS One. 2013 Nov 15;8(11):e80338. doi: 10.1371/journal.pone.0080338 (PMC3829868; doi:10.1371/journal.pone.0080338)

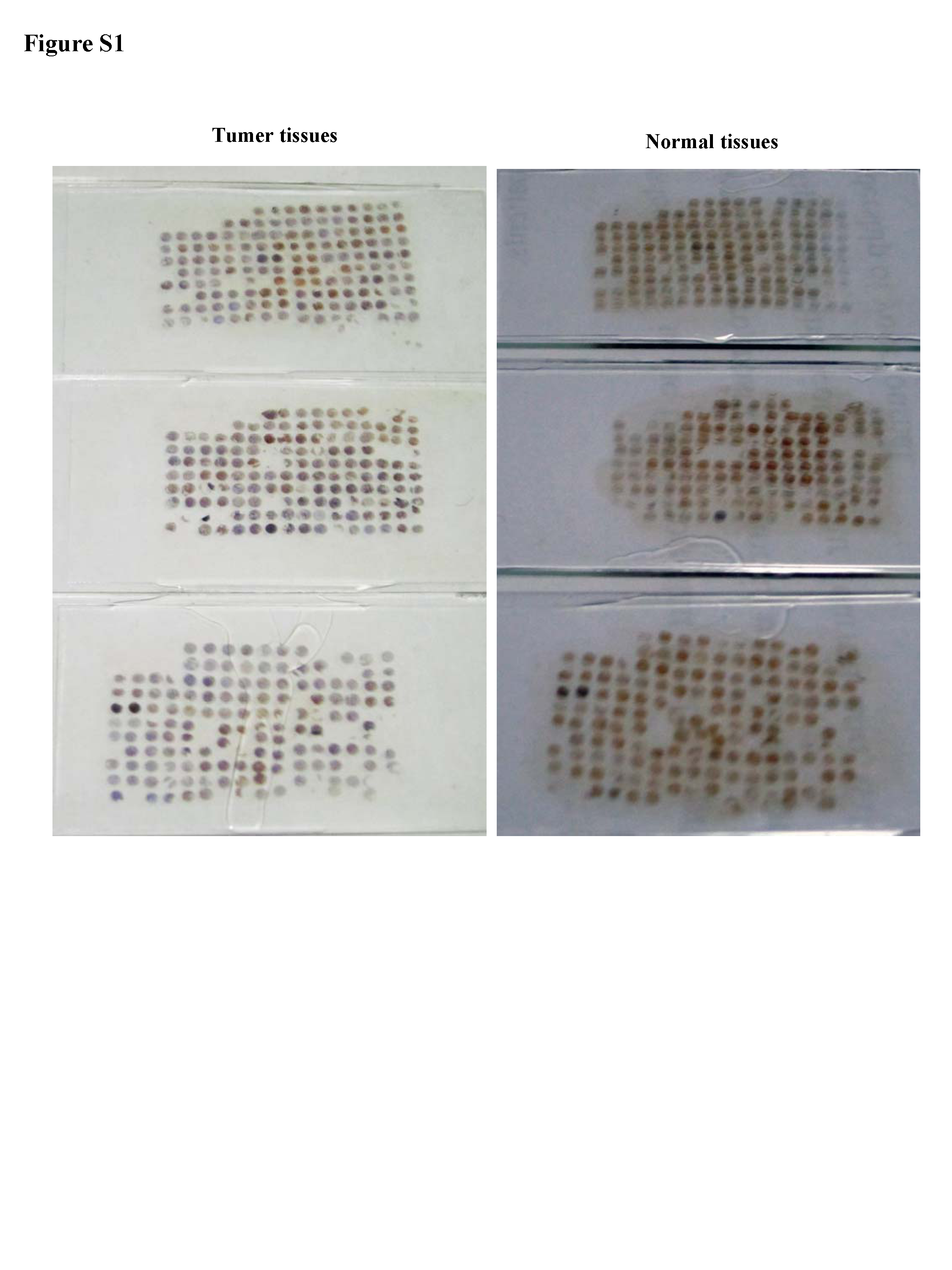

Supplement: Figure S1 — Whole picture of TMA with Beclin 1 staining. (TIF) [file pone.0080338.s001.tif]
